# Supplementary figures and images for: Cytokine and phenotypic cell profiles in human cutaneous leishmaniasis caused by Leishmania donovani
Source: PLoS One. 2023 Jan 5;18(1):e0270722. doi: 10.1371/journal.pone.0270722 (PMC9815652; doi:10.1371/journal.pone.0270722)

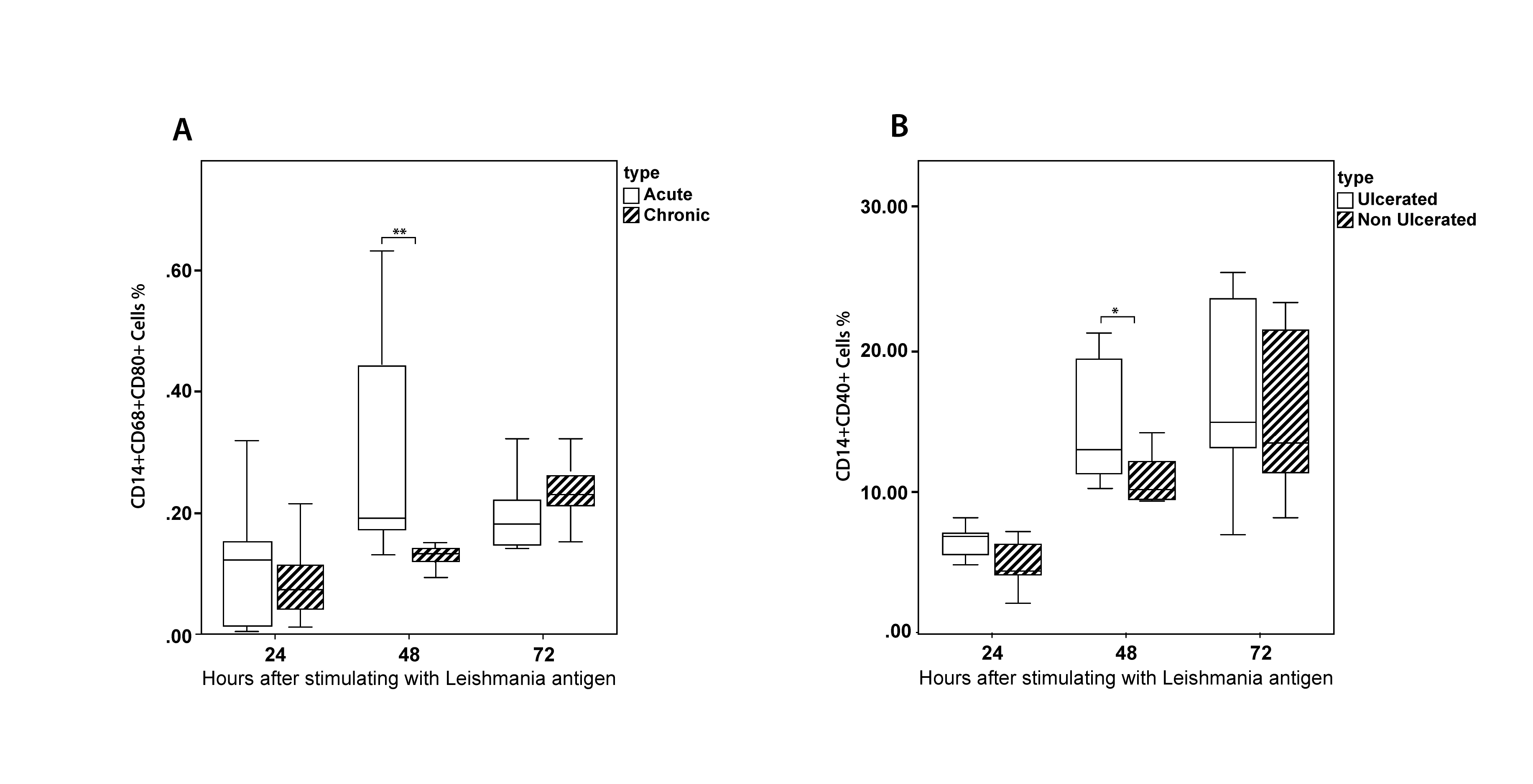

Supplement: S1 Fig — A) MI and B) M2) polarization of macrophages stimulated with SLA was compared between patients with acute (duration less than six months) and chronic lesions. The box represents the IQR (i.e. the middle 50% of the observations) with the horizontal line representing the median. The whiskers represent the main body of the data, indicating the range of the data. For statistical analysis, nonparametric Kruskal-Wallis test followed by the Dunn’s multiple comparison test was used (*p<0.05; **p<0.01; ***p<0.001). (TIF) [file pone.0270722.s001.tif]

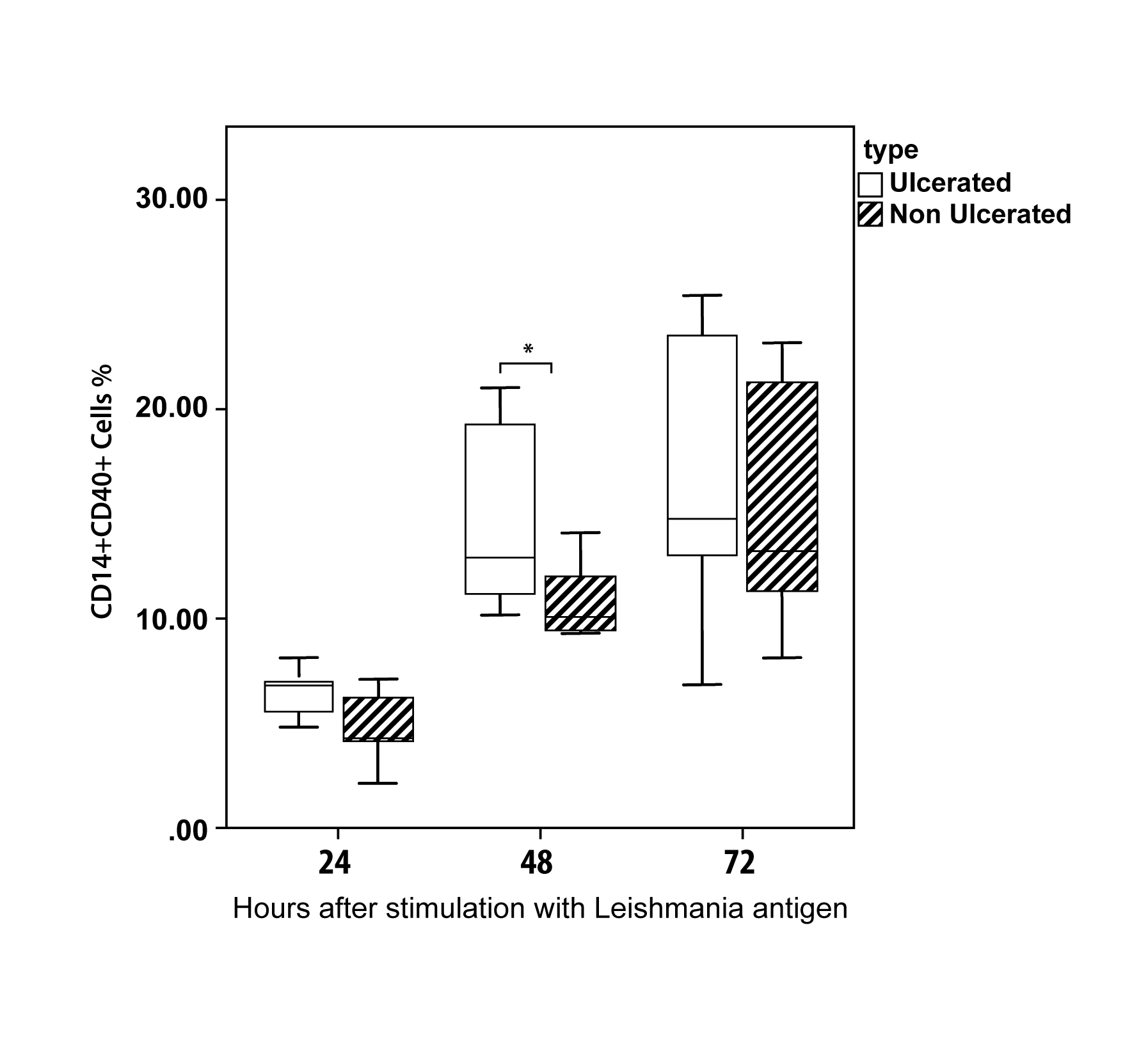

Supplement: S2 Fig — The distribution of activated macrophages following stimulation with SLA was compared between patients with ulcerated and non ulcerated lesions. The box represents the IQR (i.e. the middle 50% of the observations) with the horizontal line representing the median. The whiskers represent the main body of the data, indicating the range of the data. For statistical analysis, nonparametric Kruskal-Wallis test followed by the Dunn’s multiple comparison test was used (*p<0.05; **p<0.01; ***p<0.001). (TIF) [file pone.0270722.s002.tif]
