# Supplementary material for: Cytokine and phenotypic cell profiles in human cutaneous leishmaniasis caused by Leishmania donovani
Source: PLoS One. 2023 Jan 5;18(1):e0270722. doi: 10.1371/journal.pone.0270722 (PMC9815652; doi:10.1371/journal.pone.0270722)
Supplement: Attachment — Submitted filename: Comments.docx [file pone.0270722.s003.docx]

Although the topic is interesting, it is necessary to revise the text of the article comprehensively.

1- Abbreviations in the abstract and the main text of the article are separate. Some abbreviations have not been repeated even once in the abstract, so some abbreviations are not necessary in abstracts. For example: in line 9 Peripheral blood mononuclear cell (PBMC) and etc.
2- Keywords are not available at the end of the abstract and also the number of keywords is too high. Please remove redundant and duplicate items.
3- What was the reason for choosing cytokines? For example, why didn't you use other cytokines such as IFN-γ, IL-12, which are important in the immune response of leishmaniasis?
4- Please mention the exact time of the study in the materials and methods section in both the abstract and the main text of the article.
5- In line 100, considering the high sensitivity and specificity of the direct agglutination test (DAT), please authors explain why you did not use this test to exclude serological evidence of visceral involvement?
6- Mention the inclusion and exclusion criteria of patients and control group exactly.
7- Has this profile been analyzed and discussed in endemic and non-endemic areas?
8- In line 145 please specify how many times the cytokine assay by using ELISA was repeated? Duplicate or triplicate?
9- Please correct all the captions of the figures and the captions of the tables.
10- Overall the methodology is not well sectioned in the abstract and the main text of the article, and this sectioning should also be included in the results.
