# Supplementary material for: Cytokine and phenotypic cell profiles in human cutaneous leishmaniasis caused by Leishmania donovani
Source: PLoS One. 2023 Jan 5;18(1):e0270722. doi: 10.1371/journal.pone.0270722 (PMC9815652; doi:10.1371/journal.pone.0270722)
Supplement: Attachment — Submitted filename: Response to reviewers.docx [file pone.0270722.s004.docx]

| **Comment** | **Response** |
| --- | --- |
| **Reviewer 1** | |
| 1. The macrophage and Dendritic cells protocols are much different from conventional standard protocols. The authors need to characterize each differentiated cell type by flow cytometry before any experiment based on these cell types could be further evaluated. | We acknowledge that the ideal procedure would have been to characterize each cell type by flowcytometry.  However, plate adherent methods was used as per methodology described in previous other similar studies conducted for similar purposes, identifying macrophages by adherence morphology as we have done. Some of these references are indicated below.  The differentiation to macrophages can be further enhanced by adding M-CSF. However, we did not use these factors in this instance as they are also considered to influence polarizing of the macrophages, which was one of the evaluations done in this study. The reference by Selvapandiyan et al has been added to the methods under generation of monocyte derived macrophages (Line 143).  Selvapandiyan, A., Debrabant, A., Duncan, R., Muller, J., Salotra, P., Sreenivas, G., Salisbury, J. L., & Nakhasi, H. L. (2004). Centrin gene disruption impairs stage-specific basal body duplication and cell cycle progression in Leishmania. The Journal of biological chemistry, 279(24), 25703–25710. <https://doi.org/10.1074/jbc.M402794200>  Ty, M. C., Loke, P., Alberola, J., Rodriguez, A., & Rodriguez-Cortes, A. (2019). Immuno-metabolic profile of human macrophages after Leishmania and Trypanosoma cruzi infection. PloS one, 14(12), e0225588. <https://doi.org/10.1371/journal.pone.0225588>  Bonyek-Silva, I., Nunes, S., Bastos, R., Lima, R., Barbosa, L., Grimaldi, G., Rocha, V., Soares, M., Veras, P., de Menezes, J., Brodskyn, C., & Tavares, N. (2021). Obtainment of Macrophages from Human Monocytes to Assess Leishmania braziliensis Infection Rate and Innate Host Immune Response. Journal of visualized experiments : JoVE, (174), 10.3791/62555. https://doi.org/10.3791/62555  Furthermore, macrophages were verified by flowcytometry at the stage of checking for polarization. CD14, CD68, CD80 & CD163 markers were used to determine the macrophage M1 & M2 differentiation. CD68 is known as a marker common in both M1 & and M2 type macrophages. M1 type macrophages were identified by CD14+, CD68+, CD80+ markers and M2 macrophage cells were identified using CD14+, CD68+, and CD163+ (Line 171).  Assays of cytokine production by dendritic cells were also based on flowcytometry which involved surface staining for a dendritic cell marker followed by intra cellular staining (Line 180).  Therefore, authors believe there is adequate evidence presented in the manuscript identification of the cell types. |
| 2. CD11c alone is not enough for mature human DC characterization. | We accept that use of multiple markers would have complimented the identification of dendritic cells. CD11c was selected as one of the most widely used defining markers for DCs. Our protocol included using recombinant human granulocyte macrophage colony stimulating factor (GMCSF) and 100ng/ml recombinant IL-4 which improves differentiation of monocytes separated by adherence to dendritic cells.  The methodology used for DC identification has been well-tested and documented previously. A few examples of such other publications where CD11c has been used are mentioned below:   - Basu, A., Chakrabarti, G., Saha, A., & Bandyopadhyay, S. (2000). Modulation of CD11C+ splenic dendritic cell functions in murine visceral leishmaniasis: correlation with parasite replication in the spleen. Immunology, 99(2), 305–313. https://doi.org/10.1046/j.1365-2567.2000.00939.x - Maroof, A., & Kaye, P. M. (2008). Temporal regulation of interleukin-12p70 (IL-12p70) and IL-12-related cytokines in splenic dendritic cell subsets during Leishmania donovani infection. Infection and immunity, 76(1), 239–249. https://doi.org/10.1128/IAI.00643-07 |
| 3. You have not tested the previous exposure history in endemic controls which is necessary to be tested. | This is commonly done using the Leishmanin skin test. The antigen used in the available Leishmanin tests are considered to be incompatible with the local parasite. More importantly, this is not licensed for use in Sri Lanka by the local drug regulatory authority due to safety concerns. This is included in the discussion (Line 460) |
| 4. It is not clear why do macrophages derived from monocytes should produce Nitric oxide in the absence of any IFN-g cytokine? the same way why do authors expect IL-12 production from DCs in the absence of any stimulatory cytokine from DC? is SLA stimulation potent enough to direct IL-12 expression without any inducing signals from PRRS? These results were much more realistic if designed with whole live parasite infection. | Use of Soluble Leishmania Antigen (SLA) as an alternative to infection with live parasites is a commonly used approach in studies on immune responses in leishmaniasis, to mimic in vivo conditions. Even though it is understood that the similarity is not absolute, this crude antigen preparation is considered to consist of many antigenic components of the parasite.  An immune response by the stimulated cells without the addition of other stimulatory cytokines/mediators is also well recognized while it can be much more enhanced by addition of such.  Example use of SLA for antibodies and cytokines:   - Magalhães, A., Carvalho, L. P., Costa, R., Pita, M. S., Cardoso, T. M., Machado, P., Carvalho, E. M., Arruda, S., & Carvalho, A. M. (2021). Anti-Leishmania IgG is a marker of disseminated leishmaniasis caused by Leishmania braziliensis. International journal of infectious diseases : IJID : official publication of the International Society for Infectious Diseases, 106, 83–90. https://doi.org/10.1016/j.ijid.2021.02.016 - Coelho, E. A., Tavares, C. A., Carvalho, F. A., Chaves, K. F., Teixeira, K. N., Rodrigues, R. C., Charest, H., Matlashewski, G., Gazzinelli, R. T., & Fernandes, A. P. (2003). Immune responses induced by the Leishmania (Leishmania) donovani A2 antigen, but not by the LACK antigen, are protective against experimental Leishmania (Leishmania) amazonensis infection. Infection and immunity, 71(7), 3988–3994. https://doi.org/10.1128/IAI.71.7.3988-3994.2003 - Bottrel, R. L., Dutra, W. O., Martins, F. A., Gontijo, B., Carvalho, E., Barral-Netto, M., Barral, A., Almeida, R. P., Mayrink, W., Locksley, R., & Gollob, K. J. (2001). Flow cytometric determination of cellular sources and frequencies of key cytokine-producing lymphocytes directed against recombinant LACK and soluble Leishmania antigen in human cutaneous leishmaniasis. Infection and immunity, 69(5), 3232–3239. https://doi.org/10.1128/IAI.69.5.3232-3239.2001   Similarly, studies have shown production of IL-12 by DCs even in absence of other stimuli even though it can be much more enhanced by exogenous stimuli.  A reference where SLA has been used without other mediators is given below:   - Jawed, J. J., Majumder, S., Bandyopadhyay, S., Biswas, S., Parveen, S., & Majumdar, S. (2016). SLA-PGN-primed dendritic cell-based vaccination induces Th17-mediated protective immunity against experimental visceral leishmaniasis: a crucial role of PKCβ. Pathogens and disease, 74(5), ftw041. https://doi.org/10.1093/femspd/ftw041 |
| 5. It is not clear why monocyte derived macrophages should turn into M1 macrophages after SLA stimulation? | During the innate immune response itself, the micro environment influenced by parasite antigens and specific host responses can influence whether macrophages would assume a pro or anti-inflammatory response. M1/ M2 polarization has also been shown to vary with parasite species.  Identifying such polarization in the context of local host-parasite interactions was one of the objectives.  As mentioned in the discussion, other mediators of the innate response eg: pattern recognition receptors like TLRs on macrophage cell surface would also influence the outcome.  While T helper response would enhance macrophage polarization/activation the focus of this study was to identify how initial differential polarization of macrophages is affected in the local host-parasite context. Therefore, markers which would indicate M1 or M2 polarization were analysed.  Polarization to M1 was a finding.  Following are some publications which report the same approach to assess immune responses –i.e. cells stimulated by SLA instead of parasites without any other stimulatory mediators:   - Botana, L., Matía, B., San Martin, J. V., Romero-Maté, A., Castro, A., Molina, L., Fernandez, L., Ibarra-Meneses, A., Aguado, M., Sánchez, C., Horrillo, L., Chicharro, C., Nieto, J., Ortega, S., Ruiz-Giardin, J. M., Carrillo, E., & Moreno, J. (2018). Cellular Markers of Active Disease and Cure in Different Forms of Leishmania infantum-Induced Disease. Frontiers in cellular and infection microbiology, 8, 381. <https://doi.org/10.3389/fcimb.2018.00381> - Fernández, L., Carrillo, E., Sánchez-Sampedro, L., Sánchez, C., Ibarra-Meneses, A. V., Jimenez, M. A., Almeida, V., Esteban, M., & Moreno, J. (2018). Antigenicity of Leishmania-Activated C-Kinase Antigen (LACK) in Human Peripheral Blood Mononuclear Cells, and Protective Effect of Prime-Boost Vaccination With pCI-neo-LACK Plus Attenuated LACK-Expressing Vaccinia Viruses in Hamsters. Frontiers in immunology, 9, 843. <https://doi.org/10.3389/fimmu.2018.00843> - Carvalho, N. B., de Oliveira Prates, F. V., da Silva, R. C., Dourado, M., Amorim, C. F., Machado, P., Pacheco, F. G., Corte, T., Machado, P., Santos, D. S., & de Carvalho, E. M. (2017). In Vitro Immunomodulatory Activity of a Transition-State Analog Inhibitor of Human Purine Nucleoside Phosphorylase in Cutaneous Leishmaniasis. Journal of immunology research, 2017, 3062892. https://doi.org/10.1155/2017/3062892 |
| 6. Finally the whole discussion must compare the results from this study with the same studies based on MoDC or MoMQ stimulated with parasite antigens. | While appreciating the merits of the reviewer’s suggestion, authors would like request to strictly maintain the focus on the question that this study aimed to address, i.e. the innate immune signature in the local context which determines this clinical outcome of LCL by L. donovani.  Therefore, while adding the requested type of references we have retained references which describe PBMC responses as well as studies where the response has been measured following infection with parasites.  Following references were added (Ref 40 & 42)   - Petritus, P. M., et al. (2012). "Leishmania mexicana Induces Limited Recruitment and Activation of Monocytes and Monocyte-Derived Dendritic Cells Early during Infection." PLoS Neglected Tropical Diseases 6(10): e1858. - Maksouri, H., et al. (2017). "Moroccan strains of Leishmania major and Leishmania tropica differentially impact on nitric oxide production by macrophages." Parasites & vectors 10(1): 506. |
| Reviewer 2 | |

| 1. Please add new references in the introduction sections.  Also add a paragraph about all causative agents of CL in the world such as L. major, L. tropica, and so on.  In addition, please add more details about Th1/Th2 and its mechanisms in CL.  Use the following articles:  1- "Arginase activity of Leishmania isolated from patients with cutaneous leishmaniasis." Parasite immunology 39.9 (2017): e12454.  2- "Case report: First coinfection report of mixed Leishmania infantum/Leishmania major and human immunodeficiency virus–acquired immune deficiency syndrome: report of a case of disseminated cutaneous leishmaniasis in Iran." The American journal of tropical medicine and hygiene 98.1 (2018): 122.  3- "First case report of atypical disseminated cutaneous leishmaniasis in an opium abuser in Iran." Revista do Instituto de Medicina Tropical de São Paulo 60 (2018).  4-. "Sambucus ebulus extract stimulates cellular responses in cutaneous leishmaniasis." Parasite Immunology 41.1 (2019): e12605.  5- "Super infection of cutaneous leishmaniasis caused by Leishmania major and L. Tropica to Crithidia fasciculata in Shiraz, Iran." Iranian Journal of Public Health 48.12 (2019): 2285.  6- "Arginase/nitric oxide modifications using live non-pathogenic Leishmania tarentolae as an effective delivery system inside the mammalian macrophages." Journal of Parasitic Diseases 45.1 (2021): 65-71.  7- "Immunogenic properties of empty pcDNA3 plasmid against zoonotic cutaneous leishmaniasis in mice." Plos one 17.2 (2022): e0263993. | Added to the Introduction – Refs 9-13.  Also added to the Discussion – Ref 60.  Added. Line 43-47.  More details added in relation to the innate immune response (which is the focus of this study) which may induce different T helper responses (Refs 11-13).  None of the references are very relevant to the specific subject area addressed by this study. Therefore, while new references were added (please see above), the particular references which were suggested were not added to the manuscript.  A brief description has been added to each reference to elaborate this further.   1. <https://pubmed.ncbi.nlm.nih.gov/28731592/>   Investigates arginase activity in L major promastigotes. Nothing on human immune response.   2. <https://www.ncbi.nlm.nih.gov/pmc/articles/PMC5928724/>   Case report of DCL in HIV infected person due to L. major/L. infantum  3.<https://www.scielo.br/j/rimtsp/a/JLgQ7PPPWKwsMFW9fDq4Hpd/?lang=en>  A case report of atypical DCL due to L major in an immune-compromised person  4. https://pubmed.ncbi.nlm.nih.gov/30472741/  Report of anti-leishmania effects of a plant extract.  5. https://www.ncbi.nlm.nih.gov/pmc/articles/PMC6974865/  Describes CL due to L major and L tropica in Iran with phylogenetic analysis. Nothing on immune responses.  6. https://www.ncbi.nlm.nih.gov/pmc/articles/PMC7921220/  A delivery system to modify macrophage responses using effects of chemokine CXCL10 as an example (we did not study any chemokines)  7- <https://journals.plos.org/plosone/article?id=10.1371/journal.pone.0263993>  Evaluates the potency of the empty bacterial pcDNA3. Study is on CL in mice caused by L major. |
| --- | --- |
| 2. Please add ethics approval code in the manuscript. | Added. Line 100. |
| 3. Line 92: Add complete information regarding the following sentences “Department of Parasitology, Faculty of Medicine, University of Colombo” such as city and country. | Added. Line 100. |
| 4. Please explain completely the mentioned section: “Preparation of soluble Leishmania antigen (SLA)”. | Section on SLA preparation revised. Line 123 -133. |
| 5.Please update your L. donovani parasites cell culture section to reflect the passage number of the cells | Revised. Please see Line 127. |
| -6.Which phase of L. donovani did you use for parasite culture? Stationary or logarithmic? | Late log phase. Please see Line 128. |
| 7.Please add more information for this section “Cytokine and nitric oxide assays” and add the following articles for cytokine and nitric oxide assessment.  "Immunogenic properties of empty pcDNA3 plasmid against zoonotic cutaneous leishmaniasis in mice." Plos one 17.2 (2022): e0263993. "Arginase/nitric oxide modifications using live non-pathogenic Leishmania tarentolae as an effective delivery system inside the mammalian macrophages." Journal of Parasitic Diseases 45.1 (2021): 65-71. | We have written in the standard format when these assays are performed using commercially available kits and given the kit details for reference. |
| 8. In discussion section please add more details about Th1/Th2 and its mechanisms in CL. | More details added in relation to the innate immune response (which is the focus of this study) which may induce different T helper responses (Line 483-490). |
| Reviewer 3 | |
| 1- Abbreviations in the abstract and the main text of the article are separate. Some abbreviations have not been repeated even once in the abstract, so some abbreviations are not necessary in abstracts. For example: in line 9 Peripheral blood mononuclear cell (PBMC) and etc. | Use of abbreviation corrected in main text. Eg: consistent use of PBMC, LCL |
| 2- Keywords are not available at the end of the abstract and also the number of keywords is too high. Please remove redundant and duplicate items. | Keywords are submitted separately according to the submission guidelines for authors.  Since a limit has not been specified seven keywords have been submitted according to MESH terms as follows:  Cutaneous leishmaniasis; Leishmania donovani; innate immune response; cytokine profile; macrophage; dendritic cells; cell phenotype  There are no duplicate terms in the above list. |
| - What was the reason for choosing cytokines? For example, why didn't you use other cytokines such as IFN-γ, IL-12, which are important in the immune response of leishmaniasis? | A selected number of cytokines were decided upon based on literature review, coverage of both pro and anti inflammatory cytokines as well as availability of funds.  IL-12 production by dendritic cells is part of this study. Please see Line 255-267. |
| 4- Please mention the exact time of the study in the materials and methods section in both the abstract and the main text of the article. | Added.  Abstract – Line 15.  Methods – Line 103. |
| 5- In line 100, considering the high sensitivity and specificity of the direct agglutination test (DAT), please authors explain why you did not use this  test to exclude serological evidence of visceral involvement | We do not have an optimized DAT available in our laboratory. It is also not available in any other laboratory in the country which conducts diagnostic testing for leishmaniasis. Hence in addition to a clinical examination for signs of visceralization, rK39 immunochromatographic testing was carried out in all patients as mentioned in the text. |
| 6- Mention the inclusion and exclusion criteria of patients and control group exactly. | Already mentioned under the section on “Study locations, participants and sample collection”. Please see Line 103-121. |
| 7- Has this profile been analyzed and discussed in endemic and non-endemic areas? | The patients were all from endemic areas. The controls were recruited from endemic and non endemic areas and these two groups were analysed separately and presented. All the relevant discussions also refer to both these groups separately.  Please see Figures 1-3, 6,7. |
| 8 - In line 145 please specify how many times the cytokine assay by using ELISA was repeated? Duplicate or triplicate? | In duplicate. This was already indicated under relevant figure descriptions. It is now also added to the text.  Please see Line 168. |
| 9- Please correct all the captions of the figures and the captions of the tables. | Captions are indicated in text according to author guidelines. |
| 10- Overall the methodology is not well sectioned in the abstract and the main text of the article, and this sectioning should also be included in the results. | Description of Methods was reorganized in the abstract (Line 9-15) and main text (Line 159-192) to indicate the different sections better.  We would like to retain the sub-headings of the Results section as it is. We believe the way it is organized makes it easier for the reader to understand the significance/implications of the results. |
